# Prenatal screening for psychosocial risks in a high risk-population in Peru using the KINDEX interview

**DOI:** 10.1186/s12884-016-0799-x

**Published:** 2016-01-22

**Authors:** Andria Spyridou, Maggie Schauer, Martina Ruf-Leuschner

**Affiliations:** Center of Excellence for Psychotraumatology, Department of Psychology, Clinical Psychology & Behavioral Neuroscience Unit, University of Konstanz, Post Box D 23, D-78457 Konstanz, Germany; vivo international, http://www.vivo.org

## Abstract

**Background:**

Prenatal stress and other prenatal risk factors (e.g. intimate partner violence) have a negative impact on mother’s health, fetal development as well as enduring adverse effects on the neuro-cognitive, behavioral and physical health of the child. Mothers of low socio-economic status and especially those living in crime-ridden areas are even more exposed to a host of risk factors. Societies of extreme violence, poverty and inequalities, often present difficulties to provide adequate mental health care to the most needed populations. The KINDEX, a brief standardized instrument that assesses 11 different risk factors was used by midwives to identify pregnant women at-risk, in a suburban area with one of the highest levels of domestic violence in Lima. The instrument was designed to be used by medical staff to identify high-risk child-bearing women and, based on the results, to refer them to the adequate psychological or social support providers. The aim of this study is to assess the feasibility of psychosocial screening using the KINDEX in a Latin American Country for the first time, and to explore the relationship of the KINDEX with thee major risk areas, maternal psychopathology, perceived stress and traumatic experiences.

**Methods:**

The study was conducted in cooperation with the gynecological department of a general hospital in a suburban area of Lima. Nine midwives conducted interviews using the KINDEX of ninety-five pregnant women attending the gynecological unit of the hospital. From these, forty pregnant women were re-interviewed by a clinical psychologist using established instruments in order to assess the feasibility of the prenatal assessment in public health settings and the relationship of the KINDEX with maternal perceived stress, psychopathology symptoms and trauma load during pregnancy.

**Results:**

We found high rates of risk factors in the examined pregnant women comparable with those found in the general population. Significant correlations were found between the KINDEX sum score and the three risks areas, stress, psychopathology and trauma load as assessed in the Clinical Expert interviews. The different risks assessed by the KINDEX are related to higher levels of stress, psychopathology and trauma load, depending on the risk.

**Conclusions:**

The relationship between past adverse experience and current stressors with perceived maternal stress, psychopathology symptoms and traumatic experiences confirm the importance of prenatal assessment for psychosocial risks. The use of KINDEX by midwives providing obstetrical care to pregnant women in urban Peru is feasible and can be used to identify high-risk women and refer them to the adequate mental health or social services for necessary attention and support. Early interventions are essential to mitigating the adverse effects of maternal stress, trauma and psychopathology on the fetus and child.

## Background

Evidence-based studies of the past decades demonstrate the link between psychosocial factors in the prenatal period and adverse perinatal, neonatal and child outcomes and mother-child relationship [1–3]. The most efficient way to prevent these outcomes is to apply screening procedures during pregnancy, to identify high-risk women [4, 5].

Mother’s adverse experiences during childhood have also been linked to later mother-child relationship and offspring adjustment issues [6, 7]. A series of risk factors have been identified in the prenatal period affecting the neonate and the child later on, through epigenetic pathways [8, 9]. History of *childhood maltreatment and abuse* of the mother has been related to posttraumatic stress symptoms and depression during pregnancy and the postpartum period [10]. Children exposed to parental trauma, are also more prone to early traumatic experiences, such as emotional abuse and neglect; experiences that are related to the development of post-traumatic stress disorder (PTSD) in adulthood [11].

Childhood maltreatment has an intergenerational character. In Peru 70–80 % of the adults maltreated in childhood also maltreat their children and 41 % of the women are themselves victims of intimate partner violence (IPV) [12]. UNICEF reports for the year 2011 that punishment through physical violence is integrated in the practices of child-rearing in Peru with 41 % of parents recurring to physical punishment of their children [13]. Whereas sexual abuse against female children and adolescents, a prevalence of 19.5 % is reported for the year 2009, nevertheless only 30–40 % of the cases are denounced; prevalence is considerably higher in low SES and socially excluded areas [14] .

*Childhood victimization* has been associated to *adolescent pregnancy* [15]. Adolescent pregnancies are associated to adverse birth, and child outcomes; not only because of the young age per se, but due to all the related factors surrounding it, such as socioeconomic disadvantage [16], gynecologic immaturity, poor prenatal care, poverty and more obstetrical problems [17]. Children of teenage mothers can also present developmental delays [18]. In the metropolitan area of Lima, the prevalence of *adolescent pregnancy* in the age range of 12–14 is 0.5 % and in the age range of 15–19 an 11.7 % accounts for the total percentage of pregnancies of all age ranges for the year 2011 [12].

*IPV* during pregnancy, is also associated with a series of obstetric complications, such as kidney or urinary tract infections, high-blood pressure or edema, prenatal delivery and adverse neonatal outcomes such as low birth weight (LBW) and infant intensive care [19]. IPV is more frequent among women who have suffered childhood maltreatment or have a history of other adverse childhood experiences [20]. Interpersonal trauma exposure due to childhood adverse experiences or IPV by the mother has been found to have adverse effects on prenatal attachment [21].

*IPV* prevalence against pregnant women in Peru presents one of the highest prevalence among ten countries as elucidated in the latest report of the WHO on violence against women [22]. A study in Lima revealed a lifetime prevalence of IPV among pregnant women (physical, sexual, or emotional) of 45.1 %; prevalence of reported physical, emotional and sexual IPV was 34.2, 28.4, and 8.7 %, respectively; older (≥ 30 years), unmarried, employed, and economically disadvantaged women and those with lower education were more likely to experience lifetime and pregnancy IPV [23]. A different study in Lima with 2167 women found that women suffering physical abuse had 1.63-fold increased risk for unintended pregnancy while the risk was 3.31-fold higher among women who experienced both physical and sexual abuse compared with non-abused women [24].

Women suffering IPV during pregnancy are more likely to present depressive symptoms throughout the entire gestation and up to one year postpartum [25]. *Depression* during pregnancy has been associated with adverse birth and neonate outcomes [1, 26] while these might be worse when depression and anxiety are comorbid [27, 28]. Generally, mental health problems during pregnancy and the first years of life may increase the risk for adverse child development [29].

A recent study in Lima investigated the relationship between childhood abuse and lifetime IPV; findings indicate that any type of childhood abuse was associated with 2.2-fold increased odds of lifetime IPV, women who reported both physical and sexual abuse had 7.14-fold lifetime risk of physical and sexual IPV. The odds of experiencing physical and sexual abuse by an intimate partner in the past year was 3.33-fold higher among women with a history of childhood physical and sexual abuse as compared to women who were not abused as children [30].

In a study in Lima with 222 pregnant women carried out in 2009, prevalence of major depression was found to be as elevated as 40 %. Among married women prevalence was significantly lower (24 %); nevertheless it was more elevated among those who had not planned their pregnancy, and those who experienced pregnancy complications or presented a health problem during their pregnancy [31].

*Prenatal depression, anxiety* and *low levels of social support* have been associated with *negative prenatal attachment* that in turn predicts future mother-child attachment [32]. Additionally, women not safely attached to their fetuses in the prenatal period are more likely to indulge themselves in unhealthy behaviors such as tobacco smoking, alcohol consumption or/and drug abuse [33] exposing their fetus at even higher risk.

The adverse consequences of *substance abuse* during pregnancy on the developing fetus and their enduring effects post-partum are well-known [34–36].

In Lima, from 2001 to 2002 the prevalence of consumption of legal (alcohol & tobacco) substances in the female population was 13.4 % while for illegal substances was 0.1 %. The annual prevalence of alcohol dependence for women was found to be 2.2 and 0.5 % for tobacco [37].

*Socioeconomic factors* also play a key role in the immediate intra-uterine and general environment of the fetus. Poverty experienced early in childhood has shown to have adverse effects on children’s development, achievement and behavior [38]. In children from families of low socioeconomic status (SES) higher levels of salivary cortisol were found in comparison with their peers from higher SES; at the same time their mothers presented more feelings of depression [39, 40]. Chronic stress is more prominent in low income populations that normally have adverse living conditions such as food insecurity, increased crowding in the house and unemployment, while all these factors have been associated to LBW deliveries [41].

Among the demographic characteristics that can be a risk factor for adverse birth outcomes, are *immigration and ethnic minority status* [42]. These populations have usually undergone great social (i.e. racism) and financial pressures in such a point that ethnic differences in stress-related neuroendocrine, vascular, and immunological processes can be observed [43].

The percentage of immigration from both within Peru and foreign countries in the metropolitan area of Lima reached in 2007 34 % while the majority of these are internal migrants who were displaced from the Andean Cordillera to the big metropolitan centres seeking better life conditions and job opportunities [44]. In the latter half of the 20th century, the metropolis has grown rapidly by migration from other regions of Peru, creating human settlements similar to the *Favelas* of Brazil. These districts are characterized by lower SES and higher incidence of violence [14].

*Perceived social support* for the mother from her parents or the infant’s father has been associated with lower levels of stress in pregnancy and especially in the postnatal period [45]. Women who receive more support during pregnancy will have better nutrition and health habits, less depression symptoms and eventually better pregnancy outcomes [46, 47]. Adolescent mothers often lack of a supportive social context*,* since frequently they are single mothers and face a significant financial strain [47].

In Peru, disparities are found in the percentage of *single mothers*, depending on the residential area. The prevalence in urban areas for the age range 12–14 reached 42.2 % while for the age range 15–19 rates dropped to 21 % [44].

Finally, *medical risks and complications during pregnancy* have been associated with higher levels of stress [48, 49] and in the worse cases may lead to miscarriage [50].

Considering the severe impact of all these factors on mother’s health and fetus/child’s development we carried out this study in a developing country where all the previous factors are frequently observed in the general population and especially in low SES populations [14]. The study was carried out in one of the most crime-ridden districts of the metropolitan area of Lima. Peru, like many countries in Latin America, has increased incidence of violence against both women and children, due to cultural reasons and a complex social structure rooted in the experience of miscegenation, patriarchal violence due in part to the legacy of colonialism and the civil wars that have had disastrous consequences on human lives [51]. In this social climate women and children are the most vulnerable, exposed to daily stressful experiences of violence [14].

Prevention and early intervention in high-risk populations may be the key to avoiding adverse outcomes in child-development [52], therefore applying screening approaches as early as possible – ideally during pregnancy – is necessary to identify high-risk pregnant women and to foster a positive parent–child-relationship and positive child development [5, 53]. We explored the current literature on psychosocial risks’ assessment during pregnancy using screening instruments in the public health sector in Peru. To increase the possibilities of retrieving information we used both Spanish and English keywords but we found no published studies so in this area.

The KINDEX, a brief and easy to apply screening instrument that assesses eleven psychosocial risk factors, was developed by Schauer and Ruf-Leuschner and validated in Germany [54, 55], and has been adapted in Spanish and Greek through validation studies carried out in Spain [56] and Greece [57]. Similar assessment tools have used in other countries such as the ALPHA Form [58, 59] and the Antenatal Risk Questionnaire (ANRQ) [60], we provide a comprehensive comparison between the KINDEX and these instruments in a previous publication about the KINDEX Spanish Version [61]. In collaboration with the gynecological department of the general Hospital “Maria Auxiliadora”, in Lima, we carried out this study using the KINDEX Spanish Version for the first time in a Latin American country.

The aim of this study is two-fold:

- First, we examine the feasibility of the KINDEX in a public health setting serving low socio economic status (SES) population in a Latin American country.
- Second, we examine the relationship between the risk factors assessed by the KINDEX with the related global scores of maternal psychopathology, perceived stress and trauma load.

We also hypothesized that women scoring higher rates in the KINDEX would also present higher rates in the three global scores since the KINDEX is developed based on the current literature on psychosocial risk factors including but not limited to stress, maternal psychopathology and traumatic experiences of the mother.

## Methods

### Translation and adaptation procedure of the KINDEX

The translation procedure was done following the World Health Organization guidelines for translation process and adaptation of instruments [62]. The validation of the instrument in Spanish was carried out in Spain during a corresponding study [56]. A panel of translators, psychologists and obstetrics from Peru reviewed the Spanish version to assure that the overall content was well adapted to the local dialect. Upon review, the Spanish version of the KINDEX was accepted as is.

### Time and place of the study

The study was carried out in the hospital Maria Auxiliadora in the Southern area of Lima, between the districts of San Juan de Miraflores, Villa Maria del Triunfo and near the remote district of Villa el Salvador between March and August of 2011. Interviews were carried out in three different units of the gynecological department. The majority (85.3 %) were carried out in the external consultation unit where women attended their regular doctor’s appointment during gestation, 13.7 % in the psychoprofylaxis – maternal classes and one was carried out in the inpatient unit where women of high-medical risk were hospitalized. The Clinical Expert (CE) interviews were carried out in a private room provided by the gynecological department with no one else present during the interview apart from the interviewer and the participant. No significant difference was found in the KINDEX sum score between participants who were interviewed in the different hospital units [*H*(2) = 1.77; *p* = .41].

### Interviewers

KINDEX: All interviews (*n* = 95) were carried out by nine midwives who provided obstetrical care in the gynecological department of the hospital using the KINDEX. None of the midwives reported problems during the study with the interview procedure or the content of the interview. No dropouts from the study were registered for the midwives.

CE Interview: All CE interviews were carried out by one Spanish-speaking PhD-student of the Department of Clinical Psychology of the University of Konstanz. The interviewer was blind regarding the KINDEX assessment before the CE interview to avoid any bias. The PhD-student (from this point on: *researcher)* was trained in all standardized instruments at the Center of Excellence for Psychotraumatology at the University of Konstanz, Germany.

### Procedure

Contact with the gynecological unit of the General Hospital was established through the Peruvian Society of gynecology and obstetrics. All necessary documents including the instruments to be used were submitted to the Ethics Board of the hospital in order to receive ethical clearance. Ethical approval for the study was also given by the Ethics Committee at the University of Konstanz 1.

The coordinator of the study explained the aims of the study and presented the KINDEX to the midwives, a brief protocol for the use of the KINDEX was also available to the midwives who were asked to follow the instructions when applying the KINDEX. Among the guidelines given, were the strict randomization strategies^2^ to be followed in order to avoid selection bias when, due to time constraints, it was not possible to ask all pregnant women to participate. Participation requirements included having completed the 16^th^ week of gestation and having good comprehensive skills of Spanish. We applied this criteria in order to avoid drop-outs from the study due to the high levels of nauseas and vomiting, an often obstetric syndrome on the first three months of gestation [63, 64] that could potentially compromise the attendance to the scheduled appointments. Interviewers had to use the KINDEX to interview the participants and not to administrate it as a self-report questionnaire to the pregnant women. The KINDEX paper-pencil version is designed to be used as an interview by midwives or obstetricians and not as a self-assessment. This is the same assessment method applied in the Spanish Validation study carried out in Spain [56]. Prior to the interview the midwife had to inform the pregnant woman about the aim of the study, confidentiality and voluntary nature. Afterwards, the participant was asked to read the information sheet and give her written informed consent to be able to proceed with the interview. During the interview, the participant was in a private room where no other family members or partner was allowed. Throughout the entire screening procedure the *researcher* of the Department of Clinical Psychology of the University of Konstanz was reachable and had weekly meetings with the group of midwives collaborating in the study. A randomized subsample of forty participants was interviewed again by the *researcher* using standardized instruments to assess stress, psychopathology and trauma load.

### KINDEX

The KINDEX was developed at the University of Konstanz, Germany in 2009 [55] based on current literature on risk factors for healthy child development. Thirty-four items that assess risk factors from 11 areas that compose the KINDEX. Table 1, shows all the risk areas and the items included in each area. A detailed description of the KINDEX is found in the Spanish Validation carried out in Granada [56]. The questionnaire concludes with an open question concerning mother’s wishes for support during pregnancy and for the future with the baby. Common answers were related to child’s health, or improvement in the financial state of the family, or the possibility to receive more support for the partner.

**Table 1:** Overview of the risk factors, scales, number of items and risk definition

	*Risk Factor*	*Number of Items*	*Scale*	*Definition as a risk*	*Items included in the KINDEX Sum Score*
1	Age	1	Ordinal	≤ 21	1
2	Migration	2	Binary	Immigration mother or father	2^a^
3	Single Parent	1	Binary	Single parent	1
4	Financial problems	2	Binary	Worry about financial problems	2
			Binary	Housing index ≤ 0.5 (rooms/person)	
5	Physical Symptoms, complications, medical risks	3	Binary	Physical Symptoms, complications, medical risks	3
6	Prenatal Attachment	5	Binary	Unplanned Pregnancy	5
			Ordinal	Concerns 7–10 (Mother and Father) Joy 0–3 (Mother and Father)	
7	Perceived Stress	4	Ordinal	Stress ≥ 12	1^b^
8	Traumatic Experiences during childhood	2	Binary	Physical Abuse Sexual Abuse	2
9	Intimate partner Violence	4	Binary	Fighting increase; vociferous fights in the past 8 weeks; fisticuffs in the last 8 weeks; violence in a previous relationship.	4
10	Substance Abuse	6	Binary	Nicotine, Alcohol, Drugs/mother and father.	5^c^
11	Mental Illness	4	Binary	Ever-psychiatric diagnosis, inpatient treatment, psychotropic drugs, asked for help	4

Note: ^a^ none of the mothers or father were immigrants, ^b^none of the women Stress ≥ 12, ^c^none of the participants was consuming illicit drugs

 The internal consistency, the external validity and the criterion-concurrent validity of the KINDEX Spanish Version were proven and Cronbach’s alpha calculated for the KINDEX Spanish version was α = .67 [56]. From the reliability analysis three items were excluded because they had zero variance: the immigrant status of mother and father (2 items), drug consumption of the mother (1 item) and the Perceived Stress Scale index (PSS-4) (which is calculated summing up the four items that constitute the PSS-4) because none of the participants had a sum score higher than 12 (cut-off for high-perceived stress). The analysis therefore for the Peruvian sample consisted of 29 variables. Cronbach’s α was .66 in the present sample. Detailed description of the risk factors assessed by the KINDEX is shown in Table 1.

### Clinical expert interview

The CE interview consisted of different standardized instruments and half-standardized tools. Sociodemographic information was collected through open questions created to assess age, working situation of parents, marital status, previous and current pregnancy as well as self-reported health condition of the participant.

The standardized questionnaires used by the expert clinical psychologist to interview the participants are briefly described below:

Assessment of traumatic events and post-traumatic stress symptoms was done using the Posttraumatic Stress Diagnostic Scale (PDS) [65]. It consists of 49 items and it is divided in four parts. Part 1 consists of a short checklist, which identifies potentially traumatizing events experienced by the respondent. In part 2, respondents rate their response to this event at the time of its occurrence to determine whether the DSM IV criteria are met. In part 3 PTSD symptoms severity is rated through 17 items from 0 ("not at all or only one time") to 3 ("5 or more times a week/almost always"). Part 4 assesses interference of the symptoms with all-day functioning. The PDS yields a total symptom severity score (ranging from 0 to 51) that reflects the frequency of the 17 symptoms of PTSD in the last month. The Spanish Version of the PDS that was previously used in a study with Mexican Population [66] was used in our study. The PDS demonstrated high internal consistency (Cronbach’s *α* = .85) for our study’s sample.

The Checklist of Family Violence (CFV) [66] to assess childhood experiences of violence within the family. The same questionnaire was previously used in the study carried out in Spain. The questionnaire consists of five subscales that assess *physical abuse, verbal-emotional abuse, sexual abuse, witnessed violence* and *neglect during childhood*. The scores for each scale are obtained by summing across items and then all the scales’ scores were summed up to calculate the overall sumscore. The CFV demonstrated high reliability (Cronbach’s *α = .83*) in our study’s sample.

For the assessment of anxiety and depression, the Spanish version of the Hopkins Symptom Checklist 25 (HSCL-25) (provided by the Harvard Program in Refugee Trauma [67]) was used. It consists of 25 items: Part I of the HSCL-25 has 10 items for anxiety symptoms; Part II has 15 items for depression symptoms. The scale for each question includes four categories of response (“Not at all,” “A little,” “Quite a bit,” “Extremely,” rated 1 to 4, respectively). Two scores are calculated: the total score is the sum of all 25 items, while the depression score is the average of the 15 depression items. The validity of the instrument is well established and there is evidence for good test-retest reliability for anxiety (*r* = .75) and depression (*r* = .81). Information on internal consistency are at *α = .84* for anxiety in depression and *α = .86* [67]. Cronbach’s alpha for our study was calculated and alpha was *α* = .68.

We used the somatization scale of the Symptoms Checklist-90-R (SCL-90-R, Spanish Version) [68]. The subscale consists of 12 items rated on a 5-point Likert scale, ranging from 0 = not at all, to 4 = extremely. The score is calculated by summing across the 12 items, possible scores can range from 0 to 60. Several studies have demonstrated the reliability and validity of the SCL-90-R [68, 69]. Cronbach’s alpha calculated for the sample of our study was *α* = .75.

*Stress* was assessed through the Perceived Stress Scale (PSS-14) [70]. The PSS-4 was also used in a previous study with pregnant women in Spain [56]. The items are related to the last month. PSS-14 scores are obtained by reversing the scores on the seven positive items and then summing up all 14 items that are rated on a 5-point Likert scale ranging from 0 = never to 4 = very often. Possible scores range from 0 to 56. The 14-item version has good validity and test-retest reliability (*r* = .85), and internal consistency of Cronbach (α = .84) [70]. Cronbach´s alpha for our study’s sample was *α = .81*.

In addition to the PSS-14, the Everyday Stressors Index (ESI), [71] was used. The ESI consists of 20 items on a 4-point Likert scale ranging from 0 (not bothered at all) to 3 (bothered a great deal). A composite score of everyday stressors is calculated by summing up all items. Possible scores range from 0 to 60. The ESI assesses the areas of financial concerns, congestion, job problems, child rearing and interpersonal conflicts. As the ESI was originally created in English, in this study we used a validated version in Spanish, used in a previous study (Hopenhayn, 2010, unpublished thesis provided by the author). For the sample of this study the internal consistency of the PSS-14 was calculated and Cronbach’s *alpha was α = .84*.

In Table 2 we present all the means (*m*), ranges (*min-max*), standard deviations (*SD*) of all the measures described above.

**Table 2:** Means, (±SD) of the sample in the variables assessed in the Clinical Expert interview

*Scale*	N	*M*	*SD*	*Mdn*	*Min*	*Max*
PDS-Symptoms	40	6.97	8.91	2.0	0	29.0
Depression	40	11.97	6.58	11.0	3	28.0
Anxiety	40	7.10	4.76	6.5	0	16.0
SCL-90-R-Somatization	40	13.15	6.94	11.0	0	29
Global Psychopathology	40	2.28	3.87	1.03	−2.98	10.69
PDS-Events	40	3.45	1.90	3.5	0	8.0
CFV	40	10.65	5.44	10.0	1	24.0
Global Trauma load	40	.00	1.0	.036	−1.77	2.27
PSS-14	40	29.85	5.82	29.0	17	45
ESI	40	39.07	11.0	35.5	23	60
Global Stress	40	1.59	1.44	1.25	−1.52	4.82

Note: N (number of participants), M (mean), SD (standard deviation), Min (score minimum), Max (score maximum), CFV (checklist of family violence), PSS-14 (perceived stress scale-14 items), ESI (everyday stress index)

### Sample

Ninety-five pregnant women with an average age of 26.5 years (*SD* = 7.8, range = 14–43) constituted the sample of this study. The average gestation age was 31.5 weeks (*SD* = 5.2, range = 18-41). All participants and the respective fathers were born in Peru. The sample is described in detail, through the information collected by the KINDEX interview, in Table 3. All the participants that gave their informed consent after detailed information about the study and the interview participated throughout the entire interview with the KINDEX. From the total 95 participants 40 were invited to participate in the CE interview. No dropouts were registered. No data are available on how many women refused to participate after the information on the study was given.

**Table 3:** Overview of the risk factors in the KINDEX. Participants’ description and group comparisons of the risks between group who participated only in the KINDEX interview and the group that participated in the CE interview

*Load Factors*	*Item*		*KINDEX Mum Screen (N = 95)*	*CE Yes (N = 40)*	*CE No (N = 55)*	*P*
Gestational Age	Month of Pregnancy	M (SD)	31.53 (5.19)	28.75 (5.44)	33.55 (3.96)	.007
Age	Age in Years	M (SD)	26.52 (7.84)	26.80 (4.14)	26.13 (8.79)	ns
	Risk age ≤ 20	N (%)	26 (27.4)	15 (15.8 %)	11 (11.6 %)	ns
Migration	Mother	N (%)	0	0	0	ns
	Father	N (%)	0	0	0	ns
Single Parent	Not living with the father	N (%)	27 (28.4 %)	12 (12.6 %)	15 (15.8 %)	ns
Financial Worries	Housing index ≤ 0,5 (Room/Person)	N (%)	47 (49.5 %)	19 (20 %)	28 (29.5 %)	ns
	Financial Worries	N (%)	48 (50.5 %)	25 (26.3 %)	23 (24.2 %)	ns
Physical Complaints and Medical risk factors	Physical Complaints	N (%)	57 (60 %)	27 (28.4 %)	30 (31.6 %)	ns
	Complications	N (%)	38 (40 %)	13 (13.7 %)	25 (26.3 %)	ns
	Medical Risk Factors	N (%)	34 (35.8 %)	14 (14.7 %)	20 (21.1 %)	ns
Prenatal Bonding	Unplanned Pregnancy	N (%)	59 (62.1 %)	23 (24.2 %)	36 (37.9 %)	ns
	Joy Mother (0 to 10)	M (SD)	7,66 (2,32)	9.05 (1.70)	8.91 (1.54)	ns
	Worries Mother (0 to 10)	M (SD)	6,02 (2,71)	6.60 (3.30)	6.40 (3.25)	ns
	Joy Father (0 to 10)	M (SD)	9,18 (1,52)	7.63 (3.62)	7.82 (3.03)	ns
	Worries Father (0 to 10)	M (SD)	5,28 (3,09)	5.17 (3.55)	6.47 (3.21)	ns
Stress	PSS-4 Sum Score	M (SD)	6.44 (2.54)	6.0 (2.37)	6.76 (2.63)	ns
Abuse in Childhood	Physical Maltreatment	N (%)	41 (43.2 %)	22 (23.2 %)	19 (20 %)	ns
	Sexual Abuse	N (%)	20 (21.1 %)	10 (10.5 %)	10 (10.5 %)	ns
Intimate Partner Conflict and Violence	Increase in Conflicts (past 8 weeks)	N (%)	28 (29.5 %)	13 (13.7 %)	15 (15.8 %)	ns
	Vociferous Conflicts (past 8 weeks)	N (%)	28 (29.5 %)	9 (9.5 %)	19 (20 %)	ns
	Physical Violent Conflict (past 8 weeks)	N (%)	8 (8.4 %)	2 (2.1 %)	6 (6.3 %)	ns
	Ever violent intimate partner relationship	N (%)	21 (22.1 %)	9 (9.5 %)	12 (12.6 %)	ns
Nicotine, Alcohol and Drugs	Smoking (pregnant)	N (%)	3 (3.1 %)	2 (2.1 %)	1 (1.1 %)	ns
	Alcohol (pregnant)	N (%)	2 (2.1 %)	0 (0 %)	2 (2.1 %)	ns
	Smoking (father)	N (%)	27 (28.4 %)	9 (9.5 %)	18 (18.9 %)	ns
	Alcohol (father)	N (%)	29 (30.5 %)	9 (9.5 %)	20 (21.1 %)	ns
	Drug consumption (father)	N (%)	2 (2.1 %)	0 (0 %)	2 (2.1 %)	ns
Psychiatric History	Ever psychiatric Diagnosis	N (%)	26 (27.4 %)	10 (10.5 %)	16 (16.8 %)	ns
	Ever Psychotropic medicine	N (%)	10 (10.5 %)	6 (6.3 %)	4 (4.2 %)	ns
	Ever inpatient psychiatric treatment	N (%)	2 (2.1 %)	1(1.1 %)	1 (1.1 %)	ns
	Ever sought psychological help	N (%)	29 (30.5 %)	15 (15.8 %)	14 (14.7 %)	ns
KINDEX	KINDEX Sum Score	M (SD)	7.63 (3.55)	7.80 (4.04)	7.51 (3.17)	ns

Note: Participation in the Clinical Expert Interview (Val Yes), Participation only in the KINDEX interview (Val No), Number of participants (N), Means (M), Standard Deviation (SD), Not Significant (ns), Significance (*P*)

### Statistical analysis

Statistical analysis was performed using the SPSS 21st Version. To examine risks’ frequency reported as registered in the KINDEX interview we performed descriptive statistics.

We compared the two groups of participants (only KINDEX interview vs KINDEX and CE interview) using *t*-test for the continuous variables and chi-square for the nominal variables and Mann–Whitney-U tests for linear variables.

The sum scores of the instruments’ scales used in the CE interview were *z*-transformed and *z* values were summed up to create the three global values. The “global stress” value was created by summing the z-score of the PSS-14 and the z-score of the ESI. To calculate the “global psychopathology” value we summed up the z-score of the somatization subscale of the SCL-90-r, the z-score of the HSCL-25 (depression and anxiety) and the z-score of the PDS-symptoms (posttraumatic symptoms). The “global trauma load” value was calculated by summing up the z-score of traumatic experiences according to the PDS event-list and the z-score of the CFV (experiences of family violence).

Afterwards we explored the normality assumption through the Kolmogorov-Smirnov normality test for the global stress, global psychopathology and global trauma load as well as for the KINDEX sum score. The K-S test results for the three values, [*D*_*(*39)_ = .(11; *p* = .19; *D*_(39)=._16; *p* = .18; *D*_(39)=._09; *p =* .20] and for the KINDEX [*D*_(39)_ = .14; *p* = .03] indicate that the normality assumption is not met; therefore we calculated Spearman’s rank (rho) correlation coefficient to define relationship between variables.

Kruskal Wallis H test between subjects was conducted to assess the effect of the hospital-unit where the interview was carried out on the KINDEX sum score.

## Results

We compared the frequencies of the variables in the KINDEX and the means differences in the different measures in the CE interview. The comparison between the two groups of participants, revealed only one significant difference in the gestational age, between the group that did only participated in the KINDEX interview (*M* = 33.55, *SD* = 3.96) and the group that participate in both the KINDEX and the CE interview (*M* = 28.75, *SD* = 4.14), [t(93) = 4.9; *p* ≤ .001]. Sample descriptions and differences in risk frequencies between the groups are presented in Table 3.

### Correlations between the KINDEX Sum score and the global stress, psychopathology and trauma load

**Fig. 1:**
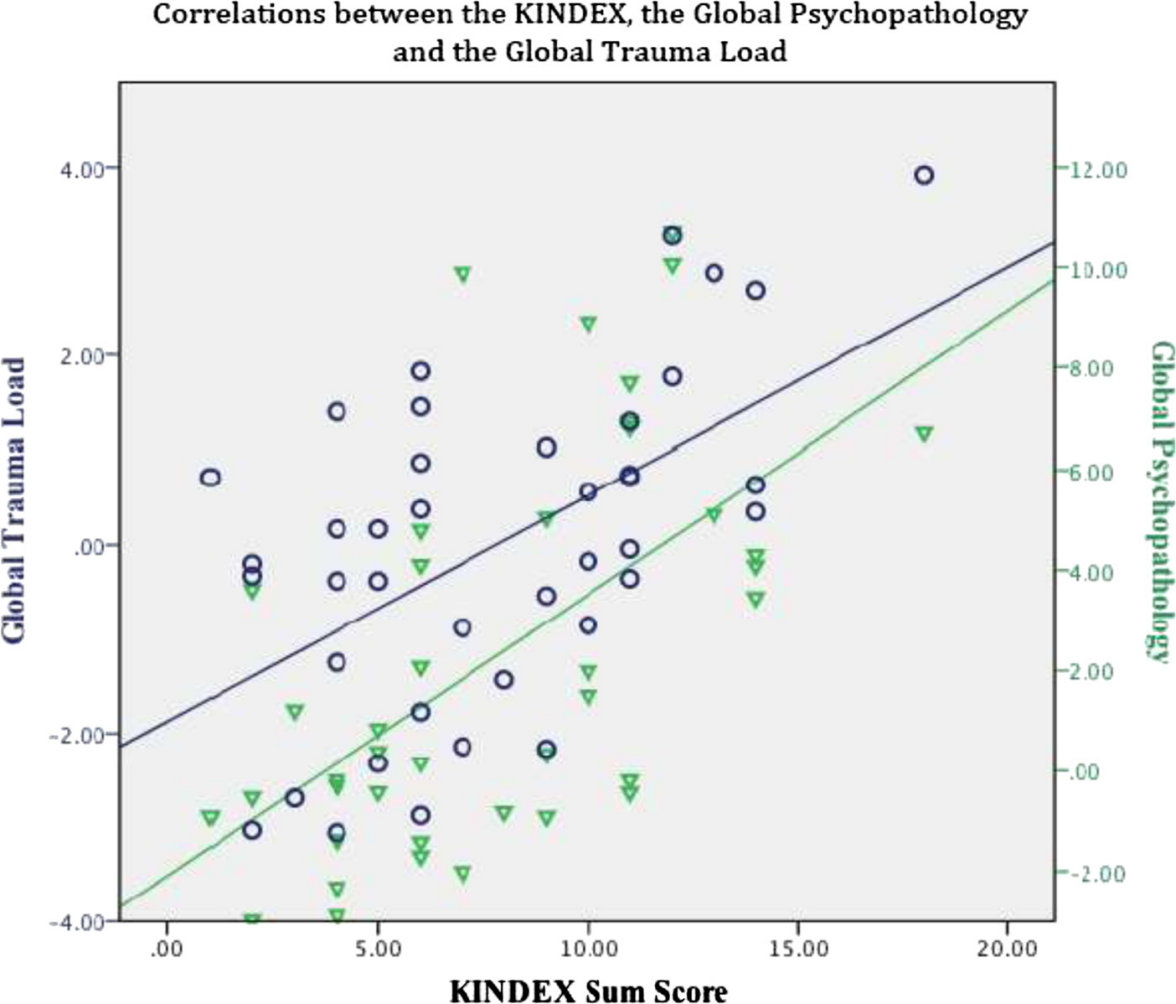
Relation between the Trauma Load (left Y axis), and the KINDEX sum score (X axis) and between Psychopathology (right Y axis) and the KINDEX sum score (X axis). The blue line shows the increasing linearity of the correlation between global trauma load and the KINDEX sum score. The green line shows the increasing linearity of the global psychopathology and the KINDEX sum score

**Fig. 2:**
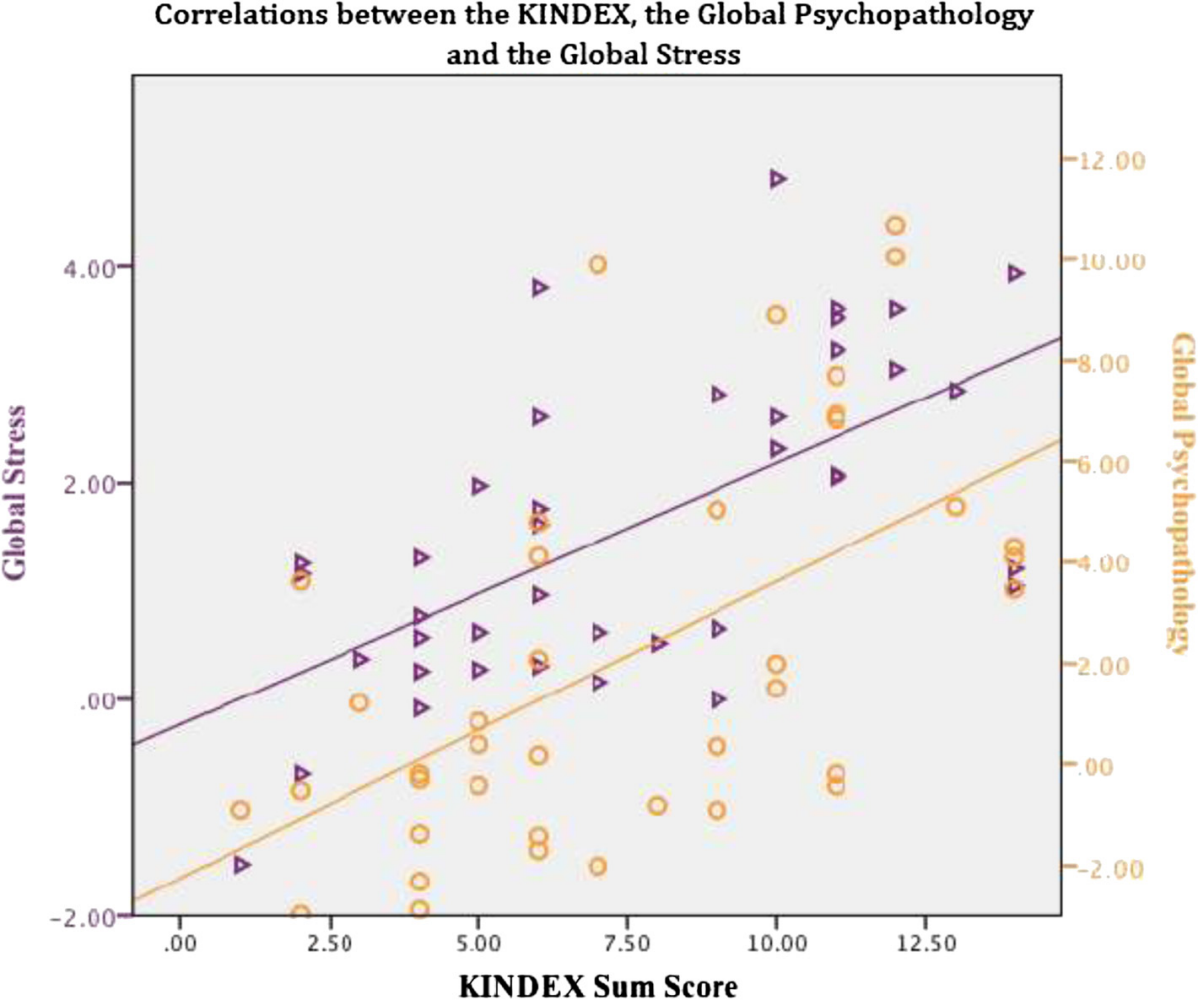
Relations between the Global Stress (left Y axis) and the KINDEX sum score (X axis) and between Psychopathology (right Y axis) and the KINDEX sum score (X axis). The purple line shows the increasing linearity of the correlation between global stress and the KINDEX sum score. The orange line shows the increasing linearity of the global psychopathology and the KINDEX sum score

We calculated the KINDEX Sum Score by summing up the 29 dichotomous items (Table 1), (*M* = 7.63, min = 0, max = 18, *SD* = 3.55). Then, we carried out correlations to examine the relations between the KINDEX, and the three risk areas assessed by the CE interview, the global perceived stress, psychopathology and global trauma load. The CE interview scales’ average, standard deviations and range are presented in Table 2. The KINDEX sum score correlated significantly with the global stress score (*r* = .62; *p* ≤ .001), the global trauma-load (*r* = .50; *p* ≤ .001) and the global psychopathology score (*r* = .61; *p* ≤ .001). In Table 4 we present the correlations between the KINDEX and the three global scores and in Fig. 1 we present in a scatter plot the correlations between Trauma Load and the KINDEX sum score and between Psychopathology and the KINDEX sum score. In Fig. 2 we present again in a scatter plot the relations Global Stress and the KINDEX sum score and between Psychopathology and the KINDEX sum score.

**Table 4:** Correlates between the KINDEX and the global stress, global psychopathology, and the global trauma load in the Clinical Expert interview

	KINDEX N = 95	Stress N = 40	Psychopathology N = 40	Trauma Load N = 40
KINDEX Sum Score	1	.62^a^	.61^a^	.50^a^
CE interview: Global Stress Score		1	.78^a^	.46^a^
CE interview: Global Psychopathology Score			1	.47^a^
CE interview: Global Trauma Load Score				1

^a^Correlation significant in the level of ≤.001

### Differences between women reporting psychosocial risks in the KINDEX and those not in relation to the global stress, psychopathology and trauma load

We examined the relationship between the risks assessed in the KINDEX in relation to the three global scores. Therefore two groups were created and comparisons were made between women who reported risks and those that had not. As shown women in Table 5, the group reporting fears for financial difficulties in the KINDEX (*n* = 25) scored significantly higher in the Global Score (*M* = 2.09; SD = 1.2) than women who reported no fears (*n* = 15), (*M* = .78, *SD* = 1.3)), [t(38) = −3.0; *p* ≤ .004]. In Table 5 all the differences between the two groups, are presented.

**Table 5:** Within Group Comparisons of the Global scores of Stress, Psychopathology and Trauma load and correlations. Group with and without risks assessed by the KINDEX items Mean (M), standard deviation (SD) and within group comparisons. Correlations between the ordinal items in the KINDEX and the three global scores

	*Global stress*							*Global psychopathology*				*Global trauma load*			
*Risk factor*	*Item*		*N*	*M (SD)*	*t (df)*	*r*	*p*	*M (SD)*	*t (df)*	*r*	*p*	*M (SD)*	*t*	*r*	*p*
Age	Age in years		40	26.5 (4.3)		-.04	ns	26.5 (4.3)		.009	ns	26.5 (4.3)		.41	.009
	Less than 20	Yes	15	1.38 (1.3)	-.68 (38)		ns	1.15 (3.1)	1.45 (38)		ns	-.86 (1.3)	2.62 (38)		.01
		No	25	1.71 (1.5)				2.96 (4.1)				.52 (1.7)			
Immigration Background	Immigration (Mother)	Yes	0	N/A				N/A				N/A			
		No	40												
	Immigration (Father)	Yes	0	N/A				N/A				N/A			
		No	40												
Social Support	Living with the father of the baby	Yes	28	1.55 (.9)	.11 (38)		ns	2.20 (2.7)	-.22 (38)		ns	.25 (1.88)	-.59 (38)		ns
		No	12	1.60 (1.6)				2.45 (4.3)				-.10 (1.67)			
Financial Difficulties	Financial Fears	Yes	25	2.09 (1.2)	−3.0 (38)		.004	3.08 (3.9)	−1.7 (38)		ns	.37 (1.87)	.124 (38)		ns
		No	15	.78 (1.3)				-.62 (3.3)				-.62 (1.27)			
	Housing Index (room/person)			.65 (.50)		-.15	ns	.65 (.50)		-.11	ns	.65 (.50)		-.33	.05
	Room/person < 0.5	Yes	19	1.81 (1.5)	-.89 (38)		ns	2.35 (4.0)	-.10 (38)		ns	.47 (1.65)	-.17 (38)		ns
		No	12	1.39 (1.3)				2.22 (3.7)				-.43 (1.71)			
Physical complaints, complications and medical risks	Physical Complaints	Yes	27	1.74 (1.5)	-.92 (38)		ns	3.0 (4.1)	-.74 (38)		ns	.14 (1.7)	-.73 (38)		ns
		No	13	1.28 (1.2)				.78 (1.6)				-.29 (1.6)			
	Complications	Yes	13	2.85 (1.2)	−4.4 (38)		≤.001	5.24 (3.8)	−3.9 (38)		≤.001	1.10 (1.5)	−3.11 (38)		.004
		No	27	1.0 (1.1)				.85 (3.0)				-.53 (1.5)			
	Medical risk factors	Yes	14	2.24 (1.4)	2.2 (38)		.03	4.48 (4.2)	−2.8 (38)		.006	.46 (1.4)	−1.26 (38)		ns
		No	26	1.22 (1.3)				1.09 (3.1)				-.25 (1.8)			
Prenatal Bonding	Unplanned Pregnancy	Yes	23	1.84 (1.5)	−1.2 (38)		ns	2.75 (3.9)	-.89 (38)		ns	.32 (1.9)	−1.42 (38)		ns
		No	17	1.25 (1.3)				1.64 (3.7)				-.44 (1.2)			
	Joy for the baby (Future mother)			8.9 (1.6)		-.07	ns	8.9 (1.6)		-.18	ns	8.9 (1.6)		-.16	ns
	Very little Joy for the baby (future mother) (≤3)	Yes	0	N/A				N/A				N/A			
		No	40												
	Concern for the future with the baby			6.4 (3.2)		.52	.001	6.4 (3.2)		.43	.006	6.4 (3.2)		.065	ns
	Very high concern about the future with the baby (future mother)	Yes	21	2.13 (1.5)	−2.5 (38)		.014	3.96 (4.1)	−3.2 (38)		.003	.06 (1.8)	-.25 (38)		ns
		No	19	1.0 (1.1)				.42 (2.4)				-.07 (.56)			
	Joy for the baby (future father)			7.74 (3.2)		-.05	ns	7.74 (3.2)		-.15	ns	7.74 (3.2)		-.17	ns
	Very little joy for the baby (future father) (≤3)	Yes	7	1.14 (.85)	.80 (38)		ns	1.70 (3.0)	.43 (38)		ns	.53 (2.1)	-.89 (38)		ns
		No	33	1.67 (1.5)				2.40 (4.0)				-.11 (1.6)			
	Concern for the future with the baby (Future father)			5.93 (3.4)		-.03	ns	5.93 (3.4)		-.09	ns	5.93 (3.4)		-.23	ns
	Very high concern about the future with the baby (future father) (≥7)	Yes	16	2.33 (1.4)	−2.9 (38)		.006	3.90 (4.0)	−2.2 (38)		.02	.35 (1.5)	−1.05 (38)		ns
		No	24	1.07 (1.2)				1.20 (3.3)				-.23 (1.8)			
Perceived stress	Stress Index			6.44 (2.54)		.59	≤.001	6.44 (2.54)		.44	.005	6.44 (2.54)		.24	ns
	Very high stress (≥12)	Yes	0	N/A				N/A				N/A			
		No	40												
Tobacco/Alcohol & Medicine/drugs	Smoking (Mother)	Yes	2	N/A				N/A				N/A			
		No	38												
	Alcohol (Mother)	Yes	0	N/A				N/A				N/A			
		No	40												
	Smoking (Future Father)	Yes	9	2.09 (1.2)	−1.1 (38)		ns	2.37 (3.5)	.27 (38)		ns	-.26 (1.3)	.52 (38)		ns
		No	31	1.42 (1.4)				1.96 (4.0)				.07 (1.8)			
	Alcohol (Future Father)	Yes	9	2.04 (1.2)	-.99 (38)		ns	2.60 (2.6)	-.28 (38)		ns	.69 (1.6)	−1.38 (38)		ns
		No	31	1.47 (1.4)				2.18 (4.1)				-.20 (1.7)			
	Drugs (Future Father)	Yes	0	N/A				N/A				N/A			
		No	40												
Violence during childhood	Physical Violence	Yes	22	1.82 (1.2)	−1.1 (38)		ns	3.66 (4.1)	−2.67 (38)		.01	.63 (1.7)	−2.79 (38)		.008
		No	18	1.31 (1.6)				.60 (2.8)				-.77 (1.4)			
	Sexual Violence	Yes	10	1.75 (1.3)	-.39 (38)		ns	3.03 (4.7)	-.70 (38)		ns	1.31 (1.5)	−3.05 (38)		.004
		No	30	1.54 (1.4)				2.03 (3.5)				-.43 (1.5)			
Intimate partner violence	Increase in fighting	Yes	13	1.65 (1.1)	-.18 (38)		ns	3.27 (3.6)	−1.12 (38)		ns	-.01 (2.2)	.041 (38)		ns
		No	27	1.56 (1.5)				1.80 (3.9)				.008 (1.4)			
	Vociferous fighting in the last 8 weeks	Yes	9	2.76 (1.2)	−2.7 (38)		.008	6.07 (2.7)	−3.90 (38)		≤.001	1.6 (1.5)	−3.73 (38)		≤.001
		No	31	1.28 (1.3)				1.18 (3.4)				-.47 (1.4)			
	Fighting with physical violence involved	Yes	2	N/A				N/A				N/A			
		No	38												
	Violence in a past Intimate relationship	Yes	9	2.01 (1.6)	-.92 (38)		ns	3.42 (4.4)	−1.02 (38)		ns	.76 (1.5)	−1.53 (38)		ns
		No	31	1.48 (1.4)				1.95 (3.7)				-.22 (1.7)			
Mental Health	Ever diagnosis of a psychiatric disorder	Yes	10	2.66 (1.0)	−2.7 (38)		.009	4.39 (2.8)	−2.07 (38)		.04	1.35 (1.4)	−3.17 (38)		.003
		No	30	1.26 (1.4)				1.58 (3.9)				-.45 (1.5)			
	Psychotropic-drugs	Yes	6	2.16 (1.4)	-.94 (38)		ns	4.58 (4.2)	−1.61 (38)		ns	1.55 (1.7)	−2.55 (38)		.01
		No	34	1.50 (1.4)				1.87 (3.7)				-.27 (1.5)			
	Ever have asked psychological help	Yes	15	2.45 (1.1)	−3.0 (38)		.004	4.90 (3.7)	−3.87 (38)		≤.001	.84 (1.7)	−2.57 (38)		.01
		No	25	1.10 (1.3)				.70 (3.0)				-.50 (1.5)			
	Ever received inpatient psychiatric treatment	Yes	1	N/A				N/A				N/A			
		No	29												

Note. *M* (mean), *SD* (standard deviation), *DF*(degrees of freedom), *t* (independent samples test t value), *p* (significance), *r* (correlation coefficient), *N* (number of participants) *N/A* not applicable, the comparisons could not be calculated when at least one of the groups was n ≤5

## Discussion

In our study untrained midwives applied the KINDEX to interview pregnant women during their daily clinical practice and in a time frame of 20 min. The midwives reported experiencing no problems throughout the study and carried out the interviews assigned to them until the conclusion of the study. In general, the involvement of the participants in the interview was satisfactory, since no dropouts were registered once the participants were involved in the study. Participants reported in the CE interview that even though the KINDEX interview was unusual they felt more cared for by the medical staff and stated that this kind of enquiries makes the medical treatment more humanized and patient-centered. As literature and our data equally show, such screenings and subsequent interventions are urgently needed considering the high prevalence rate of risk factors and the relationship of previous maternal experiences with the current perceived stress, psychopathology and trauma load.

To date, available screening instruments for psychosocial risks in the prenatal period have not been used in public health settings in Peruvian territory, even though prevalence rates indicate, that psychosocial risk is extremely prominent in this population. Our results, in accordance to the current literature, indicate that the previous maternal history is still prominent and impacts her wellbeing, influencing in a direct manner the fetus and may jeopardize the child’s development.

We expected that the KINDEX would correlate positively with the variables in the CE interview since it has been developed to identify factors that relate to psychopathology, perceived stress and traumatic load. The outcomes of our study confirmed our hypothesis and indicate that there are moderate to strong correlations between the KINDEX and the three global scores as shown in Table 4. These findings lead us to conclude that the KINDEX risk areas relate to psychopathology such as depression, to perceived stress and to traumatic experiences.

In this study we explored if the use of the KINDEX enables the midwives that work in a highly demanding context, as described above, to identify women at risk and as a consequence facilitate the referral for a more exhaustive clinical assessment by mental health specialists. As shown in a previous study we carried out in Greece using the same instrument, midwives were able to correctly identify high risk women and refer them to the mental health services [57]. We believe that it is very important to promote the use of the KINDEX in low-resources countries, such as Peru, because its use by health professionals is essential especially in settings were low-economic status and higher rates of violence set women and children at risk. Therefore, an assessment using the KINDEX could easily indicate if a pregnant woman should be referred for further specialized support or not.

As shown in other studies, stress, depression, social support and financial difficulties are often comorbid during pregnancy and pose serious problems to both mother and fetus [72, 73]. To explore further the relation of each risk factor in the KINDEX with the three global areas in the CE interview we compared the relations between the two.

In Table 5 we present the relations between the KINDEX items and risk areas assessed in the CE interview. Similarly to other studies we found that women who present traumatic experiences also have higher levels of stress and psychopathology symptoms during pregnancy [74, 75].

In this case *Adolescent pregnancy* which is considered to be a risk factor for the mother and the fetus [17], was not a factor for higher stress, or psychopathology in girls younger than 21 years in our study. Nevertheless women older than 21 years had more trauma load, explained probably by the accumulation of traumatic experiences with the passing of time.

*Social support* has been found to be a protective factor against stress during pregnancy [46]. In our study women who were not living with their partners did not have higher levels of stress, psychopathology or trauma load. Considering that a large percentage were adolescents the majority were still living with their families, and even though not living with their partners, they were probably receiving sufficient support from their parents.

*Financial difficulties* and low socio economic status are associated with increased stress that in turn affects the health of the mother and the fetus [40]. Psychiatric disorders [76] and chronic stress [41] are common and have a worse prognostic in low-income populations. In this study, women who reported having financial fears had higher levels of stress while those who were living in smaller houses reported higher levels of trauma load. This could be probably explained by the fact that these women had experienced or been exposed to more domestic violence.

*Medical complications* during pregnancy are related to a series of psychosocial adversities in pregnancy [77] while higher levels of chronic stress are linked to bio-behavioral adversities during pregnancy [78]. In our study women who had more *complications* during their pregnancy also reported higher levels of stress, psychopathology symptoms and trauma load. At the same time women who reported *medical risks* (such us gestational diabetes, or hypertension) for their pregnancy also reported higher levels of stress and psychopathology symptoms (Table 5).

Even though *negative prenatal bonding* is associated with prenatal and postnatal depression [79], in our study women that had not planned their pregnancy did not have higher levels of stress, psychopathology, or trauma load. This could be explained by the fact that many pregnancies, especially adolescent pregnancies are not planned, and are common as indicated by the high prevalence rate found in our sample (27.4 %). Nevertheless women that were more concerned about the future with the baby, and also rated their partners’ concerns as high, reported more stress and psychopathology symptoms (depression, anxiety, PTSD-symptoms) than women who were not extremely concerned.

High levels of *stress* during pregnancy have a direct impact on mother’s health and influence fetal growth and future neurocognitive development [73, 80, 81]. In this study none of the participants had extremely high stress as assessed by the PSS4 therefore comparisons between the two groups are not applicable in this case. High scores might not have been observed because of the short-version of the scale (PSS-14) that was limited only to 4 items, and might have not been able to embrace the realistic levels of stress assessed by the mother.

*Substance consumption* is linked to a series of adverse neonatal outcomes and child adverse outcomes [82]. In our sample prevalence of both maternal alcohol consumption and smoking were very low, therefore no comparisons were applicable between women who were smoking and those who were not, while none of the participants reported using illicit drugs. Between women whose partners were drinking alcohol or were smoking and those whose partners were not, no differences were found with regard to the stress, psychopathology and trauma load.

Childhood adverse experiences are associated with depression and higher levels of PTSD symptoms during pregnancy [10]. Women in this study that report having experienced physical violence in their childhood report higher levels of psychopathology and trauma load than women that did not have such experiences, confirming results of previous studies [83]. In the case of sexual abuse, women reporting such experiences in their childhood have higher levels of trauma load than women that did not report such experiences, replicating results found in other studies [83]. Even though in previous studies childhood sexual abuse has also been related to higher levels of psychopathology symptoms [6, 84] the same results were not found in our study. This might be due either to the small number of participants reporting sexual abuse participating in the CE interview (*n* = 10), or due to the existence of protective factors in adulthood, such as partners’ support.

In the present study women who had past IPV experiences did not report higher levels of stress, psychopathology or trauma load, nevertheless those women who were having vociferous fighting with their partners in the past 8 weeks did present higher levels in all three areas. This result is congruent to that found in previous studies [85], indicating that adverse partnership during pregnancy is a significant risk factor affecting the overall wellbeing of the woman, and in consequence the fetal and future child outcomes.

Studies have shown the adverse effect of depression and anxiety on pregnancy outcomes [27, 86].Women in this study that reported having previous history of psychiatric disorders, either that have received a psychiatric diagnosis or have sought help in the past, have higher levels of stress, psychopathology and trauma load. The smaller group of women (*n* = 6) that had received psychotropic drugs report higher levels of trauma load. This implies that indeed women that were previously diagnosed with a mental disorder are also in a worse position in relation to their mental health and traumatic experiences than their counterparts that were not.

We aimed to examine the generalizability of our results to the overall population from which our study’s sample was drawn. Through an exploration of the prevalence rates of the risk factors we were able to retrieve data for the general population, which revealed the similarity between the rates in the population and in our sample, confirming that our assumption of generalizability can be met.

Due to the high percentage of adolescent mothers in the Peruvian population (27.4 %) the prevalence of single mothers is also high among our sample (28.7 %) and representative of that reported rates in the general population [44].

As results indicate the prevalence of IPV in our sample (28.9 %) is similar to the rates of reported lifetime physical abuse (34.2 %) of the female population in Lima [23].

The prevalence of *childhood abuse* reported in our study was 43.2 %, a rate very similar as the one reported by UNICEF’s report in which 41 % of the parents recur to physical punishment towards their children in Peru [13],we

Regarding *sexual abuse*, reported by our sample (21.1 %) the rate is representative of the sexual abuse in the general female population in which 24 % reports that their first sexual experience was forced during adolescence or earlier [87].

Prevalence of *psychiatric history* in our study was 27.4 %, while 30.5 % had at some point asked for psychological help. These rates are similar to the ones found in the pregnant population of Lima 40 and 24 % among married women [31].

As results show the prevalence rates of *substance abuse* among the sample are not very high, nevertheless prevalence rates reported for their partners are much higher. No substance abuse rates specifically for pregnant women were found in the literature for Peru.

Our results make evident that women during pregnancy and in high-violence settings are more vulnerable in psychological suffering due to traumatic experiences, economical restrains and lack of social support among others. Psychological interventions during this period are not common even though it is well known that psychiatric disorders appear during this period [88] while affective disorders may have their onset during pregnancy or the early postpartum period [89]. In light of this information we strongly believe that psychosocial screening and targeted interventions should be applied as early as possible during pregnancy. Prevention strategies for childhood development should include psychosocial care during the gestational period and offer an integrated approach in which maternal well-being and familial functioning are taken into consideration. Such interventions should be applied and tested taking into consideration the specific needs and characteristics of each population. Unfortunately, health policies worldwide, with only a few exceptions, are still a long-way from reaching this goal.

### Study limitations

This study counts with several limitations. The instruments used in the CE interview have not been validated previously in Peruvian context although some of them have been validated in Spanish (eg. PDS, HSCL-25) some have been used in Spanish speaking countries (i.e. CFV, SCL-90-R). The KINDEX has been also validated (i.e. concurrent and external validity) in Spanish speaking population in a study in Spain, but more psychometric properties are still to be examined (i.e. construct and predictive validity). The sample size is not big enough to draw conclusions that could be generalized to all the Peruvian territory, especially in rural communities where cultural differences exist and living conditions may vary from urban regions. Larger scale studies will be needed to enable a more precise feasibility assessment in Peru. In relation to the feasibility of the use of the KINDEX we did not examine which percentage of variance is explained by the absence of dropouts contributing to the use of KINDEX. This is planned for future studies where a larger sample will be used.

## Conclusions

The feasibility of a prenatal screening in a high-risk population seems to be confirmed by two main outcomes of this study. On one hand the absence of dropouts from both the interviewers and the participants, indicating that screening for psychosocial risks is well accepted and does not interfere with the everyday obstetrical praxis even in busy hospital settings like the one of this study. The midwives did not report any difficulties during the assessment and the participants reported that they enjoyed their participation and felt more cared-for by the midwives. The prevalence rates of the majority of factors assessed by the KINDEX was well represented in our sample in comparison with the general population; even for risk areas that intimate disclosure was requested (childhood adverse experiences, IPV). This indicates that women had no difficulties in disclosing personal information to their midwives and in turn midwives addressed these questions in a manner that did not bias the response of the participants.

The relationship of the KINDEX with perceived stress, psychopathology and trauma load is demonstrated, indicating that women who score high in the KINDEX will also have more perceived stress, psychopathology symptoms and trauma load. This draws attention to the importance of referring women identified by the KINDEX as high-risk to the adequate mental health professionals, because indeed, as results show, they are in urgent need of further support.

Higher scores of stress, psychopathology and trauma were found in women reporting the presence of risk factors, confirming the results found in previous studies [90, 91]. Such experiences are often neglected and not perceived by medical staff; nevertheless they are of great importance for maternal and fetus health. In using the KINDEX, the medical staff will be able to detect such experiences and identify high-risk women.

Furthermore, longitudinal studies are needed to define the impact of the prenatal psychosocial risks on maternal and child health and maternal-child attachment and communication. Through such studies the predictive validity of the KINDEX could be examined further.

## Abbreviations

CE interview: Clinical Expert Interview
CFV: Checklist of Family Violence
ESI: Everyday Stressors Index
HSCL-25: Hopkins Symptom Checklist
IPV: Intimate partner violence
PDS: Posttraumatic Stress Diagnostic Scale
PSS-14: Perceived Stress Scale
SCL-90-R: Symptoms Checklist-90-R

## Footnotes

1 http://www.forschung.uni-konstanz.de/en/research-support/guidelines-for-proposal-writing/general-guidelines/ethics-committee/

2 On Monday the first pregnant woman, on Tuesday the second, etc.
